# The Late King Edward VII

**Published:** 1910-05-14

**Authors:** 


### THE LATE KING EDWARD VII.

### AN APPRECIATION BY ONE WHO KNEW HIM.

The tragedy of the sudden death of King Edward VII. has created the deepest grief and a feeling of something like dismay, not only amongst people of all classes within the British Empire but throughout the world. He had become, in a very real sense, not only the Beloved Father and Monarch of his own subjects, but every nation rightly regarded him as the great Peace Maker. Englishmen who are abroad at this time must have been touched by the spontaneous and general expressions of reverence for our dead King and of sympathy with the English race in their great affliction. The suddenness of the blow has fired the imagination of the whole world. Everywhere foreigners, when the news reached them, went into mourning before leaving their homes in the morning, as the writer saw all classes do in Spain, for example. Every British Embassy and Consulate was crowded on the day after his death with foreign sympathisers, who, as representatives of the peoples of the world, spontaneously desired to show their respect for a great and good King and their sympathy with his people. We have said that King Edward was properly trusted and beloved as the Father of his people; but foreigners, too, had so deep a regard for and belief in our late King that his death has everywhere been felt to be a grievous loss, and even, in a sense, a personal one. He tried to do his duty as a Monarch, and success crowned his efforts. Hence the void created by his lamented death has been felt throughout the civilised world.

### His Sympathy for the Sick.

We who have to do with hospitals and the sick will remember our late King with affection always. He has ever been mindful of the sick and the suffering. No trouble or responsibility, or output of personal time and service was too great to cause King Edward to hesitate in lending a hand whenever his aid was needed and deserved. Great as were the services he rendered to the cause of the voluntary hospitals by his prodigal output of service, time, and advocacy, these were by no means the most valuable gifts the voluntary hospitals received at his hands. It was King Edward who set an example of courage in support of good works and an insistence upon the most economical and efficient administration of our hospitals which have materially contributed to make British hospitals probably the best managed and most comfortable palaces of pain to be found in the world. To lay down a policy of wise economy, good management, and the removal of existing abuses, as King Edward did, needed the possession of no little personal courage on his part. But our late beloved King was quite equal to the demands made upon him in these respects, and his name will go down to history not only as the King Peace Maker but as the King Philanthropist, with the courage to enforce reforms wherever he was persuaded that they were necessary. All honour to the memory of the Monarch and Emperor whose first aims were to preserve peace and to minimise suffering all the world over.

### His Wise Philanthropy.

The hospital world owes much to our late Sovereign. He refused to preside at the festival dinner to Guy's Hospital until the scheme for its rebuilding and reorganisation was made adequate to its needs and traditions. So, when he did take the chair, some six months later, his action produced the largest financial result ever desired from a festival dinner. It also revolutionised the practice of this ancient method of raising funds for charity, and gave it new life and power. Again, when the fact that no adequate system of insurance, sick pay, and old-age provision for nurses was made plain, King Edward, then Prince of Wales, in conjunction with Queen Alexandra, boldly supported the Royal National Pension Fund for Nurses from its earliest foundation, so that it has become the greatest thrift fund for women workers in the world.

### His Influence on Charitable Work.

It would be impossible to set forth here the multiplicity of good which King Edward did for hospitals and charities of all kinds, but it has left its mark all over the field of charity. King Edward's crowning work for hospitals found expression in the establishment of the King's Hospital Fund. King Edward, then Prince of Wales, threw himself heart and soul into this scheme. The first meeting was held at Marlborough House, at which he took the chair. Before entering the room to preside at the meeting he paused, with his hand on the door, and said to the gentlemen to whom he had entrusted the work of initial organisation: "We shall find critics and opponents, no doubt, present to-day, but I rely upon you to meet their objections and to satisfy them, for the establishment of this Fund is very near my heart, and it is needed. We must therefore carry the scheme through to-day before I vacate the chair." We mention this as a striking example of the thought our late King put into most things he touched, and of his soundness of judgment and persistence in well-doing once he put his hand to the work. Let the King's Hospital Fund be remembered as a monument of the splendid services rendered by King Edward VII. to the cause of the sick. No monarch could desire any greater, more permanent, or better record of his whole-hearted endeavours in the days of health to minister with all his strength and wisdom to the needs of the sick and suffering. So the hospital world, like the great world outside, may thank God and take courage that King Edward VII. should have nobly lived and laboured and reigned over a free people who loved him as the Great Father of the Nation and the Peacemaker of the twentieth century.

